# Indoor and outdoor PM_10_ levels at schools located near mine dumps in Gauteng and North West Provinces, South Africa

**DOI:** 10.1186/s12889-016-3950-8

**Published:** 2017-01-06

**Authors:** Vusumuzi Nkosi, Janine Wichmann, Kuku Voyi

**Affiliations:** 1 School of Health Systems and Public Health, Faculty of Health Sciences, University of Pretoria, P.O. Box 2034, Pretoria, 0001 South Africa; 2 Environment and Health Research Unit, South African Medical Research Council, Pretoria, South Africa

**Keywords:** Mine dumps, Schools, Air pollution, Asthma, South Africa

## Abstract

**Background:**

Few studies in South Africa have investigated the exposure of asthmatic learners to indoor and outdoor air pollution at schools. This study compared outdoor PM_10_ and SO_2_ exposure levels in exposed (1–2 km from gold mine dumps) and unexposed schools (5 km or more from gold mine dumps). It also examined exposure of asthmatic children to indoor respirable dust at exposed and unexposed schools.

**Methods:**

The study was conducted between 1 and 31 October 2012 in five schools from exposed and five from unexposed communities. Outdoor PM_10_ and SO_2_ levels were measured for 8-h at each school. Ten asthmatic learners were randomly selected from each school for 8-h personal respirable dust sampling during school hours.

**Results:**

The level of outdoor PM_10_ for exposed was 16.42 vs. 11.47 mg.m^−3^ for the unexposed communities (*p* < 0.001). The outdoor SO_2_ for exposed was 0.02 ppb vs. 0.01 ppb for unexposed communities (*p* < 0.001). Indoor respirable dust in the classroom differed significantly between exposed (0.17 mg.m^−3^) vs. unexposed (0.01 mg.m^−3^) children with asthma at each school (*p* < 0.001).

**Conclusion:**

The significant differences between exposed and unexposed schools could reveal a serious potential health hazard for school children, although they were within the South African Air Quality Standards’ set by the Department of Environmental Affairs. The indoor respirable dust levels in exposed schools could have an impact on children with asthma, as they were significantly higher than the unexposed schools, although there are no published standards for environmental exposure for children with asthma.

## Background

Acute or chronic exposure to particulate matter <10 μm in diameter (PM_10_) is a worldwide concern. It is associated with the exacerbation of asthma attacks, the decline in lung function, preterm birth and an increase in hospital visits and deaths among children with pre-existing asthma conditions or respiratory diseases [1–9]. Children are the most susceptible population since they can receive a higher dose of PM_10_ in the lungs compared to adults. This may be due to greater fractional deposition with each breath and/or larger minute ventilation relative to lung size [10]. Children spend approximately 7 or more hours per day at school, mostly in classrooms. This is the second highest time spent in the indoor environment after home, so makes the school an interesting area to assess air pollution exposure [11, 12]. Children’s personal exposure to indoor air pollutants, including PM_10,_ is largely determined by pollutant concentration outdoors [13–15]. Research studies have shown that mine dumps are a major contributor to particulate matter air pollution to surrounding communities and that proximity is associated [16] with increased risk asthma symptoms. [17, 18] Taking into consideration that school children spend one-third of their total time inside school buildings, it is evident that air quality inside the classrooms should be of concern [5, 19, 20]. Whether it is indoor or outdoor, PM_10_ may have adverse biological effects when exposures are prolonged in children [21]. Children who have asthma are a group that is particularly vulnerable to airborne pollutants such as PM_10_, SO_2_ and respirable dust. [22–27] In order to estimate the risk to children, particularly those with asthma; and develop a mitigation strategy, the actual levels of these air pollutants at schools near mine dumps need to be measured.

No studies appear to have investigated whether proximity to mine dumps influences outdoor air pollution and indoor respirable dust levels in South African schools. Thus, the aim of this study was to measure levels of PM_10_ and SO_2_ outside, as well as respirable dust indoors in schools exposed and unexposed to mine dust between 1 and 31 October 2012.

This study forms part of a bigger project initiated by Mine Health Safety Council of South Africa (MHSC) around communities located near mine dumps in Gauteng and North West, provinces in South Africa.

## Methods

### Study area, study period and demographics

**Fig. 1:**
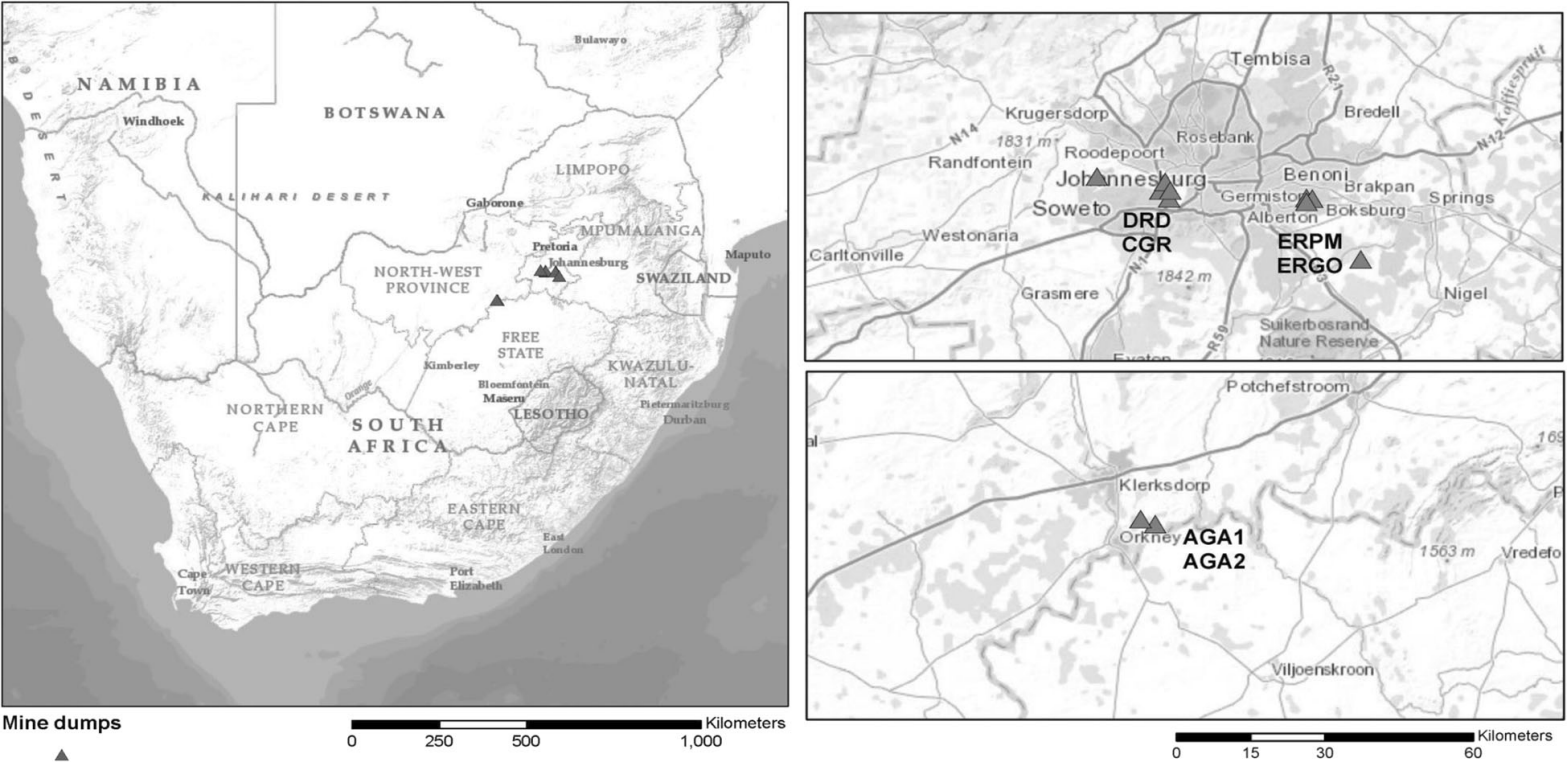
Location of mine dumps tailings in South Africa

Schools located 1–2 km (exposed) and 5 km or more (unexposed) [28, 29] from pre-selected five mine dumps in Gauteng and North West Provinces of South Africa were included in the study. The study was conducted between 1 and 31 October 2012. Table 1, lists the selected schools and Fig. 1 shows a map of the study area. The socio-economic and demographic profile of exposed and unexposed schools was similar.

**Table 1:** Ten schools selected in the study located in Gauteng and North West provinces, South Africa during 1–31 October 2012

Mine dump facility	Province	Exposed school^a^	Unexposed school^b^
Durban Roodepoort Deep (DRD)	Gauteng	Kgatelopele secondary	PJ Simelane secondary
Crown Gold Recoveries (CGR)	Gauteng	Noordgesig secondary	Job Rathebe secondary
Ergo	Gauteng	Geluksdal primary	Windmill Park primary
East Rand Proprietary Mines (ERPM)	Gauteng	Lakeside primary	Windmill Park secondary
Anglo Gold Ashanti	North West	Vaal Reefs secondary	Inkangmahlale secondary

^a^1-2 km from mine dumps

^b^5 km or more from dumps

**Fig. 2:**
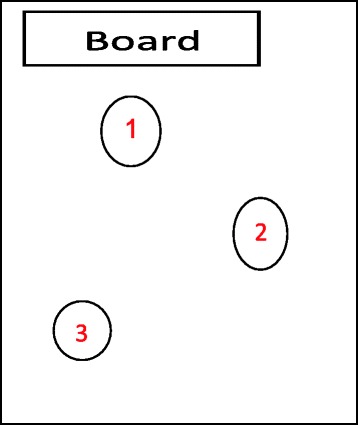
Seating positions of sampled learners

### Study participants

The study participants were 13–14-year-old asthmatic learners. Ten of these learners were selected from each of the 10 schools (5 exposed and 5 unexposed) in Gauteng and North West provinces in South Africa. The socio-economic and demographic profile of the exposed and unexposed schools was similar. They form a subset of participants in the International Study of Asthma and Allergies in Children (ISAAC), 2012 survey. Three learners in each of two classrooms and four in one classroom were purposively selected for personal air sampling; Fig. 2 shows the seating position of learners within the classroom.

### Exclusion criteria

Commuting learners and learners that were not diagnosed as having asthma by the doctor/physician were not included in the study.

### Personal air sampling

Personal air sampling was performed in the breathing zone of asthmatic learners during school hours from 8 am to 15 pm using a Gillian Personal Sampler. All the gravimetric sampling was done in accordance with the requirements of General Methods for Sampling and Gravimetric Analysis of Respirable, Thoracic and Inhalable Dust, Regulation 14/3 [30]. Respirable particulate fraction is that fraction of inhaled airborne particles that can penetrate beyond the terminal bronchioles into the gas-exchange region of the lungs, usually measured in μg.m^−3^ [31].

### Ambient air monitoring

An AEROQUAL mobile air monitoring station was used to measure the ambient PM_10_ and SO_2_ within the school premises, between 08 h00 and 15 h00, at a height of one meter, on an open space or ground. The mobile air monitoring station was placed downwind, in the South-easterly direction, where the wind is predominately blowing in the study areas.

### Statistical analyses

All statistical analyses were performed using Stata™ version 14. Respirable dust was considered as the dependent variable and ambient air pollutants such as PM_10_, SO_2_ and date of sampling were independent variables. Eight-hour mean concentration of ambient air pollutants such as PM_10_, SO_2_ and respirable dust were determined. Pearson correlations coefficients were estimated to better understand their inter-relationship of PM10, SO2 and respirable dust. Descriptive statistics were used to explain data; standard deviations, percentiles and ranges were to illustrate data as appropriate. The *t*-test was using was used to compare the mean levels of respirable dust, PM_10_ and SO_2_ of exposed and unexposed schools. Ten filters for each school were weighed in the accredited laboratory. Data from the mobile air monitoring station and the laboratory were merged for analysis.

Crude and adjusted β-coefficients and 95% confidence intervals (CI) were calculated with univariate and multiple backwards hierarchical standard regression analysis to estimate the association between of respirable dust and independent variables such as PM_10_ outdoor concentration, SO_2_ outdoor concentration, school location, the date of sampling. Independent variables with a *p*-value <0.2 obtained in the univariate regression analysis were included in the multivariable regression analysis. A *p*-value < 0.05 in the multivariate regression analysis was considered statistically significant [32]. The most parsimonious multivariate model is reported, i.e. the model with variables having a *p*-value < 0.05.

## Results

**Fig. 3:**
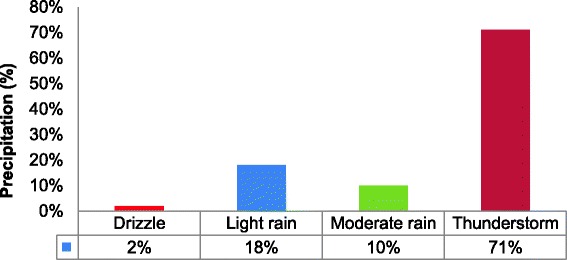
Shows the percentage precipitation during the sampling period, October 2012

A total of 100 learners’ age between 13 and 14 years took part in the study. Fifty were from the communities exposed and other fifty from the unexposed communities. October encompassed part of the wet season in South Africa, Fig. 3 shows the percentage precipitation during the sampling period [33]. The mean outdoor 8-h concentrations of PM_10_ and SO_2_ for both exposed and unexposed schools were within the South African Air Quality Standards’ set by the Department of Environmental Affairs [34]. However, there was a significantly higher 8-h mean concentration of PM_10_ (*p* < 0.001), SO_2_ (*p* < 0.001) and respirable dust (*p* < 0.001) observed in schools located near mine dumps, as compared to unexposed schools (Table 2).

**Table 2:** Distribution of the daily 8-h mean concentrations of PM_10_ and SO_2_ and indoor respirable dust in ten selected schools in the study located in Gauteng and North West provinces, South Africa between 1 and 31 October 2012

Exposed^a^	Mean ± SD	95 CI	*p*-value^c^	25th percentile	Median	75th percentile	Range
Respirable dust (μg/m^3^)	0.17 ± 0.10	(0.14–1.99)	<0.001	0.10	0.17	0.20	0.02–0.7
PM_10_ (μg/m^3^)	16.42 ± 3.67	(15.37–17.46)	<0.001	17.30	18.00	18.10	9.30–19.40
SO_2_ (ppb)	0.02 ± 0.01	(0.01–0.03)	<0.001	0.10	0.10	0.04	0.00–0.05
Unexposed^b^							
Respirable dust (μg/m^3^)	0.06 ± 0.03	(0.05–0.07)	<0.001	0.05	0.06	0.08	0.01–0.15
PM_10_ (μg/m^3^)	11.47 ± 4.90	(10.08–12.87)	<0.001	9.30	13.30	15.20	3.10–16.50
SO_2_ (ppb)	0.01 ± 0.01	(0.001–0.02)	<0.001	0.00	0.01	0.02	0.00–0.20

^a^Exposed: schools located 1–2 km from mine dumps

^b^Unexposed: schools located 5 km or more from mine dumps

^c^ *p*-values of the *t*-test

PM_10_: particulate matter <2.5 μm in diameter; SO_2_: sulphur dioxide

Table 3 shows the Spearman correlation coefficients of the indoor and outdoor pollutants. PM_10_ and respirable dust were significantly positively correlated with each other (*p* < 0.001). The strongest correlation coefficient observed was *r* = 0.41 (*p*-value = 0.02) between PM_10_ and respirable dust. No significant correlation was observed between SO_2_ and PM10, SO_2_ and respirable dust.

**Table 3:** Spearman’s correlation coefficients for outdoor PM_10_ and SO_2_ and indoor respirable dust in ten selected schools in the study located in Gauteng and North West provinces, South Africa between 1 and 31 October 2012

Pollutants	Spearman correlation coefficients		
	Respirable dust	PM_10_	SO_2_
Respirable dust (μg/m^3^)	1.00		
PM_10_ (μg/m^3^)	0.41 (<0.001)*	1.00	
SO_2_ (ppb)	0.02 (0.675)	0.29 (0.004)*	1.00

*p < 0.05; SO_2_: sulphur dioxide; PM_10_: particulate matter <10 μm in diameter

Results from the multivariate standard regression model (Table 4) indicated significant associations between respirable dust and PM_10_ (β = 0.27; 95% CI: 0.05–0.49); SO_2_ (β = −0.31; 95% CI:−0.57– − 0.05) and school location (β = −0.95; 95% CI:−1.18– − 0.71) respectively. The date of sampling was significantly associated with the indoor respirable dust in schools located near mine dumps in the univariate analysis (β = −11.59; 95% CI:−18.57– − 5.6), but not in the multivariate analysis.

**Tables 4:** Univariate and multivariate β coefficients of standard regression analysis with 95% confidence intervals of respirable dust in 10 schools located1-2 km and ≥5 km from mine dumps in Gauteng and North West provinces, South Africa between 1 and 31 of October 2012

	Univariate analysis			Multivariate analysis^a^		
	β coefficients	95% CI	*P*-value	β coefficients	95% CI	*P*-value
*Independent variables*						
PM_10_ outdoor concentration	0.56	0.31–0.80	<0.001	0.27	0.05–0.49	0.018
S0_2_ outdoor concentration	−0.31	−0.57– − 0.05	0.018	−11.59	−18.57– − 5.60	0.001
School location	−0.93	−1.15– − 0.72	<0.001	−0.95	−1.18– − 0.71	<0.001
Day of sampling	−0.11	−0.15– − 0.06	<0.001	−	−	−

^a^Model adjusted for all variables in this table, except date of sampling and number of asthmatic per school

## Discussion

The results of this study suggest that schools located near mine dumps in South African are exposed to higher levels of concentration of outdoor air pollutants such as outdoor PM_10_ and SO_2_ and indoor respirable dust compared to those located further away. Children with increased vulnerability to air pollution would be more likely to experience exacerbated asthma symptoms and attacks on both low and high air pollution days [35, 36]. The mean 8-h concentration levels of PM_10_ and SO_2_ were well below the South African Air Quality Standards’ set by the Department of Environmental Affairs [34]. However, even such low levels might have a negative impact on the respiratory health of susceptible individuals, since there is no threshold limit for pollutants to trigger asthma symptoms and attack [37]. Amenity deficiencies in schools such as poor maintenance and structural damage perhaps due to lack of funding observed during the survey may lead to pollutants infiltrating from the outdoor environment into the classrooms. Research studies have shown that asthmatic children miss more days at school than those without asthma [38–40]. This indicates that children attending schools in communities located near mine dumps, their respiratory health is not only compromised but also their academic performance might be negatively affected.

In assessing the school environment both indoor and outdoor air pollution contribution should be considered, since children often play outside their classrooms during breaks [41]. In this study, a statistically significant correlation between PM_10_ and indoor respirable dust was observed; this is in agreement with other research studies that the outdoor PM_10_ can infiltrate and influence the indoor concentration levels of respirable dust. [42–45] The exposure assessment study conducted during the dry season in one of the mine dumps included in this study showed that the average 24-h ambient air pollution levels were twenty times high than what is recommended by the South African Air Quality Standards’ set by the Department of Environmental Affairs [17, 34]. This suggest that mine dumps can have an influence on the indoor air pollution levels in the houses and schools of the nearby communities. A cross-sectional study conducted in the communities located close to mine dumps in South Africa showed that a significant number of residents still use coal or fossil fuel as the main residential heating or cooking fuel type; [18, 46] probably contributes to the ambient levels of SO_2_ in these communities. Research studies have indicated that asthmatics are very sensitive to inhaled SO_2_, and experience changes in pulmonary function and respiratory symptoms after periods of exposure to SO_2_ as short as 10 min is sufficient to induce broncho-constriction [47–50].

Limitations of the study were that only SO_2_ was determined. Other gaseous pollutants such ozone and nitrogen dioxide were not included due to the mobile air monitoring station which only had one SO_2_ sensor. Only 10 schools were included in the study, due to limited funds and Gillian personal pumps. The study had a small sample size resulting in a small statistical power and the findings of this study cannot be generalized to the whole population/schools in communities near mine dumps. The study was conducted only in spring wet season and measurements were done once per school in each community. Therefore, it is suggested that further studies should be conducted to contrast indoor and outdoor levels in dry and wet seasons for a longer duration.

## Conclusion

The significant differences between exposed and unexposed schools could reveal a serious potential health hazard for school children. The indoor respirable dust levels in exposed schools could have an impact on children with asthma, as they were significantly higher than the unexposed schools, although there are no published standards for environmental exposure for children with asthma.

## Abbreviations

μg/m^3^: Microgram per cubic meter
AGA: Anglo gold Ashanti
CGR: Crown gold recoveries
CI: Confidence intervals
DRD: Durban Roodepoort deep
ERPM: East rand proprietary mine
NRF- DAAD: National research fund – Deutscher Akademischer Austausch Dienst
PM_10_: Particulate matter with size less than 10 μm in diameter
SO_2_: Sulphur dioxide
SRA: Simple regression analysis
